# Conserved ‘Hypothetical’ Proteins: New Hints and New Puzzles

**DOI:** 10.1002/cfg.66

**Published:** 2001-02

**Authors:** Michael Y. Galperin

**Affiliations:** National Center for Biotechnology Information National Library of Medicine National Institutes of Health Bethesda MD 20894 USA

## Abstract

Conserved hypothetical proteins, i.e. conserved proteins whose functions are still unknown, pose a challenge not just to functional genomics but also to general biology. For many conserved proteins, computational analysis provides only a general prediction of biochemical function; their exact biological functions have to be established through direct experimentation. In the few cases when this has been accomplished, the results were remarkable, revealing the deoxyxylulose pathway and a new essential enzyme, the ITP pyrophosphatase. Comparative genome analysis is also instrumental in illuminating unsolved problems in biology, e.g. the mechanism of FtsZ-independent cell division in *Chlamydia, Ureaplasma* and *Aeropyrum* or the role of uncharacterized conserved domains in signal transduction.


**Comparative and Functional Genomics**
*Comp Funct Genom* 2001; **2**: 14–18.

**Conference Paper**

### Conserved 'hypothetical' proteins: new hints and new puzzles

Michael Y. Galperin*
*National Center for Biotechnology Information, National Library of Medicine, National Institutes of Health, Bethesda, MD 20894, USA*

**Correspondence to: M. Y. Galperin, National Center for Biotechnology Information, National Library of Medicine, National Institutes of Health, Bethesda, MD 20894, USA. E-mail: galperin@ncbi.nlm.nih.gov*

### Abstract

**Conserved hypothetical proteins, i.e. conserved proteins whose functions are still unknown, pose a challenge not just to functional genomics but also to general biology. For many conserved proteins, computational analysis provides only a general prediction of biochemical function; their exact biological functions have to be established through direct experimentation. In the few cases when this has been accomplished, the results were remarkable, revealing the deoxyxylulose pathway and a new essential enzyme, the ITP pyrophosphatase. Comparative genome analysis is also instrumental in illuminating unsolved problems in biology, e.g. the mechanism of FtsZ-independent cell division in *Chlamydia, Ureaplasma* and *Aeropyrum* or the role of uncharacterized conserved domains in signal transduction. Copyright © 2001 John Wiley & Sons, Ltd.**

**Keywords: sequence analysis; orthologous proteins; database; phylogenetic pattern; GGDEF domain**

The availability of more than 30 complete genomes, representing eight out of 10 main bacterial phyla (**http://www.ncbi.nlm.nih.gov/PMGifs/Genomes/new_btax.html**), two out of three classes of Archaea, and as many as four classes of eukaryotes, certainly makes one excited about the perspectives of new genome-based biology. The 'dark ages of restriction maps, unanchored cosmid contigs, and vast parts of the genome inaccessible and marked with the phrase "Here be dragons"', mentioned by Ian Dunham (2000), seem to be gone forever. The growing problem, however, is that once genomes are sequenced and potential genes predicted, we still have very little idea of what many of these genes code for, even if their products are similar to uncharacterized proteins from other genomes. Thus, 'dragons' are being increasingly replaced by the politically correct euphemism 'conserved hypothetical proteins', which comprise anywhere from 20% to 40% of proteins encoded in each new sequenced genome (Bork, 2000; Galperin and Koonin, 2000; Nelson *et al.*, 2000). Here, I briefly discuss the power and limitations of comparative genomics in deducing functions of uncharacterized proteins and offer several examples of insights that could not have been made without genome comparisons.

When an open reading frame is annotated as a 'conserved hypothetical protein', this does not necessarily mean that the function of its product is completely unknown, and even its very existence is questionable. Certain exceptions notwithstanding (Natale *et al.*, 2000), if a conserved protein is found in several genomes, it is not really hypothetical any more. Quite often, a general prediction of its function can be made based on a conserved sequence motif, subtle sequence similarity to a previously characterized protein, or presence of diagnostic structural features (Galperin and Frishman, 1999). Many 'conserved hypothetical proteins' can be confidently predicted as ATPases, GTPases, methyltransferases, metalloproteases, DNA- or RNA-binding proteins, or membrane transporters (Aravind and Koonin, 1999). This does not mean, of course, that their exact biological function is known; that can only be established through direct experimentation. A long list of such protein families, for which the general biochemical prediction could have been made but exact biological function is still elusive, can be found, e.g. in the COG

Copyright © 2001 John Wiley & Sons, Ltd.

database (**http://www.ncbi.nlm.nih.gov/COG**) functional group R (Tatusov *et al.*, 2000).

Of all the uncharacterized proteins, the most fascinating ones are those found in many distantly related organisms, including those with small genomes. For most pathogens, adaptation to the parasitic lifestyle included a drastic reduction in the genome size through the loss of genes encoding many metabolic enzymes, transcriptional regulators and membrane permeases. The genes that have been retained in at least several distantly related organisms can be expected to be essential for cell survival (Arigoni *et al.*, 1998). The uncertainty regarding their function betrays our lack of understanding of some basic aspects of cell physiology. These genes also attract a significant attention as potential targets for antimicrobial drugs (Galperin and Koonin, 1999). One such case includes a conserved family of short proteins, homologous to the *Escherichia coli* protein YbeB and plant protein Iojap, which is involved in biogenesis of chloroplast ribosomes. Members of this family are found in every sequenced bacterial genome, except for mycoplasmal ones, and are also encoded in worm and fly genomes. Although the phenotype of the *iojap* mutation has been known for years (Han *et al.*, 1992), the exact function of this protein remains enigmatic. Another interesting example involves homologues of *E. coli* protein YjeE, which comprise UPF0079 in PROSITE or COG0802 in the COG database (Table 1). Based on the conserved nucleotide-binding motif, this protein family has been annotated as 'probable ATP-binding protein' in PROSITE, 'uncharacterized P-loop hydrolase' in Pfam, and 'predicted ATPase or kinase' in COGs. Using the recently developed 'genome context' approaches (Galperin and Koonin, 2000; Huynen *et al.*, 2000) provides some additional hints. First, the phylogenetic pattern of this COG (**http://www.ncbi.nlm.nih.gov/cgi-bin/COG/palox?seq = yjeE**) shows that this protein is found in every bacterium, except for mycoplasmas. A look at other conserved proteins with the same phylogenetic pattern shows that most of such COGs (MurA, MurB, MurG, FtsI, FtsW, DdlA) represent enzymes of cell wall biosynthesis. Second, it turns out that in a number of Proteobacteria, members of this COG form conserved operons with the gene *amiB*, encoding cell wall amidase. Judging from the phylogenetic pattern and operon structure, one can suggest that this ATPase might have something to do with cell wall turnover. The determination of the three-dimensional structure of this protein confirmed the prediction that it is an ATPase but still has not resolved the problem of its biological function (A. V. Teplyakov, personal communication). It is clear, therefore, that establishing the 'real' biological function for this extremely well-conserved ATPase will require direct experimentation. Similarly, determination of the three-dimensional structures of predicted methyltransferase YibK and predicted nucleotide-binding protein MJ0577 has confirmed the general prediction of their biochemical functions, but failed to shed light on their exact biological roles (Lim *et al.*, 2000; Zarembinski *et al.*, 1998). In such cases, follow-up experimental research is needed to pinpoint the biological function.

In the few instances when the function of conserved 'hypothetical' proteins has been established, the results were quite spectacular. For example, three widely conserved proteins were identified as enzymes of the deoxyxylulose pathway of terpenoid biosynthesis (Herz *et al.*, 2000; Luttgen *et al.*, 2000; Rohdich *et al.*, 1999). The identification of ITP pyrophosphatase activity in MJ0226, a member of a conserved protein family that includes *E. coli* YggV (Hwang *et al.*, 1999), was even more illuminating. In hindsight, it seems ludicrous that such an activity had not been recognized previously. Indeed, IMP is a normal cell metabolite, serving as a precursor for both AMP and GMP in the purine biosynthesis pathway. There is a certain chance that IMP can be phosphorylated to ITP, e.g. by adenylate kinase. Since inosine can pair with adenine, thymine and cytosine, which is often used to design degenerate PCR primers, ITP would pose a tremendous mutational risk for the cell. Even IDP would be dangerous, as it can be converted to ITP in a single nucleotidyl kinase-catalysed step. It is small wonder that ITP pyrophosphatase is encoded in almost every microbial genome and also in worm, fly and human. There is little doubt that other conserved families have similarly important but so far unrecognized functions.

In other instances, the biological function of a protein is known, at least to some extent, whereas the biochemical mechanism of its action remains unclear. For example, products of *pdxA* and *pdxJ* genes of *E. coli* are believed to catalyse the ring closure step of pyridoxine biosynthetic pathway (Laber *et al.*, 1999). Recently, the product of the

Copyright © 2001 John Wiley & Sons, Ltd. 

Table 1. Phylogenetic patterns of selected protein families in complete genomes

| Species | Pyridoxine biosynthesis | | | | Cell division | | | | Signal transduction | | | Poorly characterized | | | |
|---|---|---|---|---|---|---|---|---|---|---|---|---|---|---|---|
| | *pdxA* | *pdxJ* | SNZ1 | SNO1 | *minC* | *minD* | *minE* | *ftsZ* | GGDEF | EAL | HD-GYP | *yjeE* | *ybaK* | *yibK* | *ybeB* |
| **COG number** | **1995** | **0854** | **0214** | **0311** | **0850** | **0455** | **0851** | **0206** | **2199** | **2200** | **2206** | **0802** | **2606** | **0219** | **0799** |
| Bacteria | | | | | | | | | | | | | | | |
| *Escherichia coli* | + | + | − | − | + | + | + | + | 19 | 17 | − | + | + | + | + |
| *Haemophilus influenzae* | − | − | + | + | − | − | − | + | − | − | − | + | + | + | + |
| *Pseudomonas aeruginosa* | + | + | − | − | + | + | + | + | 33 | 21 | 3 | + | + | + | + |
| *Vibrio cholerae* | + | + | − | − | + | + | + | + | 41 | 19 | 9 | + | + | + | + |
| *Xylella fastidiosa* | + | + | − | − | + | + | + | + | 3 | 3 | 1 | + | − | + | + |
| *Rickettsia prowazekii* | − | − | − | − | − | − | − | + | 1 | 1 | − | + | − | − | + |
| *Neisseria meningitidus* | + | + | − | − | + | + | + | + | − | − | − | + | + | − | + |
| *Helicobacter pylori* | + | + | − | − | + | + | + | + | − | − | − | + | − | + | + |
| *Campylobacter jejuni* | + | + | − | − | − | − | − | + | 1 | − | − | + | − | + | + |
| *Deinococcus radiodurans* | − | − | + | + | + | + | + | + | 16 | 5 | 4 | + | − | + | + |
| *Synechocystis* sp. | + | + | − | − | + | + | + | + | 23 | 13 | 2 | + | − | + | + |
| *Bacillus subtilis* | − | − | + | + | + | + | − | + | 4 | 2 | − | + | + | + | + |
| *Mycoplasma genitalium* | − | − | − | − | − | − | − | + | − | − | − | − | − | − | − |
| *Mycoplasma pneumoniae* | − | − | − | − | − | − | − | + | − | − | − | − | − | − | − |
| *Ureaplasma urealyticum* | − | − | − | − | − | − | − | − | − | − | − | − | − | − | − |
| *Mycobacter. tuberculosis* | − | − | + | + | − | + | − | + | 1 | 2 | − | + | − | + | + |
| *Borellia burgdorferi* | − | − | − | − | − | + | − | + | 1 | 1 | 1 | + | − | − | + |
| *Treponema pallidum* | − | − | − | − | − | + | − | + | 1 | − | 3 | + | − | − | + |
| *Chlamydia trachomatis* | − | − | − | − | − | − | − | − | − | − | − | + | − | + | + |
| *Chlamydia pneumoniae* | − | − | − | − | − | − | − | − | − | − | − | + | − | + | + |
| *Aquifex aeolicus* | + | + | − | − | + | + | − | + | 11 | 6 | 1 | + | − | − | + |
| *Thermotoga maritima* | − | − | + | + | + | + | − | + | 9 | − | 9 | + | − | − | + |
| Archaea | | | | | | | | | | | | | | | |
| *Methanococcus jannaschii* | − | − | + | + | − | + | − | + | − | − | − | − | − | − | − |
| *Methanobacterium thermoautotrophicum* | − | − | + | + | − | + | − | + | − | − | − | − | − | − | − |
| *Archaeoglobus fulgidus* | − | − | + | + | − | + | − | + | − | − | − | − | − | − | − |
| *Pyrococcus horikoshii* | − | − | + | + | − | + | − | + | − | − | − | − | − | − | − |
| *Aeropyrum pernix* | − | − | + | + | − | − | − | − | − | − | − | − | + | − | − |

Presence or absence of a particular protein in each complete genome was first checked using the COG database (Tatusov *et al.*, 2000) and then verified by TBLASTn against complete genome sequences using corresponding proteins from *E. coli, D. radiodurans*, and *B. subtilis* as queries, as described by Natale *et al.* (2000). Pluses and minuses indicate whether a given protein is encoded in the particular genome. The numbers indicate the total number of the domains of each kind encoded in the genome; GGDEF and EAL domains are often found on the same polypeptide chain.

SNZ1 ('snooze') gene, which comprises one of the best conserved enzymes across all three main lineages of life and was referred to previously as a 'Rosetta Stone' protein (Das *et al.*, 1997; Galperin and Koonin, 1997), and its counterpart SNO1 ('snore'; Padilla *et al.*, 1998) have been implicated in catalysing the same reaction in organisms that are devoid of PdxA and PdxJ homologues (Ehrenshaft *et al.*, 1999; Osmani *et al.*, 1999). Nevertheless, despite the critical importance of resolving the mechanism of pyridoxine biosynthesis, these enzymes remain poorly characterized.

Another example shows how little we actually understand about signal transduction in bacteria. Sequence analysis of the proteins of a two-component signal transduction system, encoded in complete bacterial genomes, revealed complex domain architectures of many of them and allowed the identification of a number of novel conserved domains (Aravind and Ponting, 1997, 1999; Galperin *et al.*, 1999; Taylor and Zhulin, 1999). While exact biochemical functions of some of these domains remain obscure, their association with various components of the signal transduction machinery indicates that they also participate in signal transduction (Aravind and Ponting, 1997,

Copyright © 2001 John Wiley & Sons, Ltd. 

1999; Galperin *et al.*, 1999). Two such domains of unknown function, referred to as GGDEF and EAL domains, respectively, have been described in putative diguanylate cyclases and phosphodiesterases, regulating cellulose synthesis in *Acetobacter xylinum* (Galperin *et al.*, 1999; Tal *et al.*, 1998). These two domains, referred to as DUF1 and DUF2 in the SMART database (Schultz *et al.*, 2000) and comprising COG2199 and COG2200, respectively, in the COG database (Tatusov *et al.*, 2000), along with the recently described phosphodiesterase-related HD-GYP domain (COG2206), have all been implicated in signal transduction based solely on the domain architectures of the corresponding proteins (Galperin *et al.*, 1999). Recently, involvement of the GGDEF domain in the control of cell differentiation of *Caulobacter crescentus* has been demonstrated experimentally (Aldridge and Jenal, 1999), providing the ultimate validation of its functional assignment. All these domains are widely represented in bacterial genomes (Table 1). Unfortunately none of the 19 *E. coli* proteins that contain the GGDEF domain or 17 *E. coli* proteins that contain the EAL domain has been ever characterized experimentally. The proteins that contain these domains in *B. subtilis* have not been studied either. Annotation of all the proteins that contain GGDEF, EAL or HD-GYP domains as 'conserved hypothetical' seems to be quite an understatement.

Finally, there are cases where we might assume that we understand the mechanism of the process and the function of a particular protein in it, only to find out later that these assumptions were poorly justified. The most striking example involves the function of the cell division proteins MinC, MinD, MinE and FtsZ. In *E. coli*, the GTPase FtsZ forms the division septum; the MinD protein catalyses ATP-dependent activation of division inhibitor MinC, which prevents septum formation everywhere except for the central plane of the cell, where it is disabled by MinE (reviewed in Rothfield *et al.*, 1999). The problem with this scheme, which became apparent only through genome comparisons (Galperin and Grishin, 2000), is the absence of these ostensibly essential genes in many of completely sequenced genomes and the presence of MinD homologues in some organisms that do not encode either MinC or MinE (Table 1). Cases like this represent an important contribution of comparative genomics to general biology. They help us understand that data obtained on model organisms (*E. coli, B. subtilis*, yeast) are not always readily transferable to all other organisms; sometimes they just reflect idiosyncrasies of these model organisms.

In conclusion, conserved 'hypothetical' proteins pose a challenge not just to functional genomics, but also to general (micro)biology. Computational methods, including sophisticated sequence analysis, phylogenetic patterns and domain fusions, and gene neighbourhoods can and should be used for prediction of the likely (biochemical) properties of these proteins. However, the ultimate biological function(s) for members of new conserved protein families can be established only through direct experimentation. As the list of the conserved 'hypothetical' proteins keeps growing, comparative genomics can help in identifying the most intriguing proteins in every genome and creating reasonable and verifiable hypotheses for their experimental analyses.

### References

Aldridge P, Jenal U. 1999. Cell cycle-dependent degradation of a flagellar motor component requires a novel-type response regulator. *Mol Microbiol* **32**: 379–391.

Aravind L, Koonin EV. 1999. Gleaning non-trivial structural, functional and evolutionary information about proteins by iterative database searches. *J Mol Biol* **287**: 1023–1040.

Aravind L, Ponting CP. 1997. The GAF domain: an evolutionary link between diverse phototransducing proteins. *Trends Biochem Sci* **22**: 458–459.

Aravind L, Ponting CP. 1999. The cytoplasmic helical linker domain of receptor histidine kinase and methyl-accepting proteins is common to many prokaryotic signalling proteins. *FEMS Microbiol Lett* **176**: 111–116.

Arigoni F, Talabot F, Peitsch M, *et al.* 1998. A genome-based approach for the identification of essential bacterial genes. *Nature Biotechnol* **16**: 851–856.

Bork P. 2000. Powers and pitfalls in sequence analysis: the 70% hurdle. *Genome Res* **10**: 398–400.

Das S, Yu L, Gaitatzes C, *et al.* 1997. Biology's new Rosetta stone. *Nature* **385**: 29–30.

Dunham I. 2000. Genomics—the new rock and roll? *Trends Genet* **16**: 456–461.

Ehrenshaft M, Bilski P, Li MY, Chignell CF, Daub ME. 1999. A highly conserved sequence is a novel gene involved in *de novo* vitamin B6 biosynthesis. *Proc Natl Acad Sci U S A* **96**: 9374–9378.

Galperin MY, Frishman D. 1999. Towards automated prediction of protein function from microbial genomic sequences. In *Methods in Microbiology*, vol. 28, Craig AG, Hoheisel JD (eds). Academic Press: London; 245–263.

Galperin MY, Grishin NV. 2000. The synthetase domains of cobalamin biosynthesis amidotransferases CobB and CobQ belong to a new family of ATP-dependent amidoligases, related to dethiobiotin synthetase. *Proteins* **41**: 238–247.

Copyright © 2001 John Wiley & Sons, Ltd. 

Galperin MY, Koonin EV. 1997. Sequence analysis of an exceptionally conserved operon suggests enzymes for a new link between histidine and purine biosynthesis. *Mol Microbiol* **24**: 443–445.

Galperin MY, Koonin EV. 1999. Searching for drug targets in microbial genomes. *Curr Opin Biotechnol* **10**: 571–578.

Galperin MY, Koonin EV. 2000. Who's your neighbor? New computational approaches for functional genomics. *Nature Biotechnol* **18**: 609–613.

Galperin MY, Natale DA, Aravind L, Koonin EV. 1999. A specialized version of the HD hydrolase domain implicated in signal transduction. *J Mol Microbiol Biotechnol* **1**: 303–305.

Han CD, Coe EH Jr, Martienssen RA. 1992. Molecular cloning and characterization of iojap (ij), a pattern striping gene of maize. *EMBO J* **11**: 4037–4046.

Herz S, Wungsintaweekul J, Schuhr CA, *et al*. 2000. Biosynthesis of terpenoids: YgbB protein converts 4-diphosphocytidyl-2C-methyl-D-erythritol 2-phosphate to 2C-methyl-D-erythritol 2,4-cyclodiphosphate. *Proc Natl Acad Sci U S A* **97**: 2486–2490.

Huynen M, Snel B, Lathe W 3rd, Bork P. 2000. Predicting protein function by genomic context: quantitative evaluation and qualitative inferences. *Genome Res* **10**: 1204–1210.

Hwang KY, Chung JH, Kim SH, Han YS, Cho Y. 1999. Structure-based identification of a novel NTPase from *Methanococcus jannaschii*. *Nature Struct Biol* **6**: 691–696.

Laber B, Maurer W, Scharf S, Stepusin K, Schmidt FS. 1999. Vitamin B6 biosynthesis: formation of pyridoxine 5′-phosphate from 4-(phosphohydroxy)-L-threonine and 1-deoxy-D-xylulose-5-phosphate by PdxA and PdxJ protein. *FEBS Lett* **449**: 45–48.

Lim K, Zhang H, Tempczyk A, *et al*. 2000. Hypothetical proteins from *Haemophilus influenzae*: two new structures implying methyltransferase function. American Crystallographical Association Meeting, St. Paul, MN; 36–37.

Luttgen H, Rohdich F, Herz S, *et al*. 2000. Biosynthesis of terpenoids: YchB protein of *Escherichia coli* phosphorylates the 2-hydroxy group of 4-diphosphocytidyl-2C-methyl-D-erythritol. *Proc Natl Acad Sci U S A* **97**: 1062–1067.

Natale DA, Galperin MY, Tatusov RL, Koonin EV. 2000. Using the COG database to improve gene recognition in complete genomes. *Genetica* **108**: 9–17.

Nelson KE, Paulsen IT, Heidelberg JF, Fraser CM. 2000. Status of genome projects for non-pathogenic bacteria and Archaea. *Nature Biotechnol* **18**: 1049–1054.

Osmani AH, May GS, Osmani SA. 1999. The extremely conserved *pyroA* gene of *Aspergillus nidulans* is required for pyridoxine synthesis and indirectly for resistance to photosensitizers. *J Biol Chem* **274**: 23565–23569.

Padilla PA, Fuge EK, Crawford ME, Errett A, Werner-Washburne M. 1998. The highly conserved, co-regulated SNO and SNZ gene families in *Saccharomyces cerevisiae* respond to nutrient limitation. *J Bacteriol* **180**: 5718–5726.

Rohdich F, Wungsintaweekul J, Fellermeier M, *et al*. 1999. Cytidine 5′-triphosphate-dependent biosynthesis of isoprenoids: YgbP protein of *Escherichia coli* catalyzes the formation of 4-diphosphocytidyl-2-C-methylerythritol. *Proc Natl Acad Sci U S A* **96**: 11758–11763.

Rothfield L, Justice S, Garcia-Lara J. 1999. Bacterial cell division. *Ann Rev Genet* **33**: 423–448.

Schultz J, Copley RR, Doerks T, Ponting CP, Bork P. 2000. SMART: a web-based tool for the study of genetically mobile domains. *Nucleic Acids Res* **28**: 231–234.

Tal R, Wong HC, Calhoon R, *et al*. 1998. Three *cdg* operons control cellular turnover of cyclic di-GMP in *Acetobacter xylinum*: genetic organization and occurrence of conserved domains in isoenzymes. *J Bacteriol* **180**: 4416–4425.

Tatusov RL, Galperin MY, Natale DA, Koonin EV. 2000. The COG database: a tool for genome-scale analysis of protein functions and evolution. *Nucleic Acids Res* **28**: 33–36.

Taylor BL, Zhulin IB. 1999. PAS domains: internal sensors of oxygen, redox potential, and light. *Microbiol Mol Biol Rev* **63**: 479–506.

Zarembinski TI, Hung LW, Mueller-Dieckmann HJ, *et al*. 1998. Structure-based assignment of the biochemical function of a hypothetical protein: a test case of structural genomics. *Proc Natl Acad Sci U S A* **95**: 15189–15193.

Copyright © 2001 John Wiley & Sons, Ltd.
